# Morphology, biochemistry and connectivity of Cluster N and the hippocampal formation in a migratory bird

**DOI:** 10.1007/s00429-022-02566-y

**Published:** 2022-09-17

**Authors:** Dominik Heyers, Isabelle Musielak, Katrin Haase, Christina Herold, Petra Bolte, Onur Güntürkün, Henrik Mouritsen

**Affiliations:** 1 AG Neurosensorik, Institute of Biology and Environmental Sciences, Carl Von Ossietzky University Oldenburg, 26111 Oldenburg, Germany; 2 Research Centre for Neurosensory Sciences, Carl Von Ossietzky University Oldenburg, 26111 Oldenburg, Germany; 3 Medical Faculty, Cécile and Oskar Vogt- Institute of Brain Research, University Hospital and Heinrich-Heine University Düsseldorf, 40225 Düsseldorf, Germany; 4 Institute for Cognitive Neuroscience, Department of Biopsychology, Faculty of Psychology, Ruhr-University Bochum, 44780 Bochum, Germany

**Keywords:** Migratory bird, Magnetoreception, Wulst, Hippocampal formation, Avian

## Abstract

The exceptional navigational capabilities of migrating birds are based on the perception and integration of a variety of natural orientation cues. The “Wulst” in the forebrain of night-migratory songbirds contains a brain area named “Cluster N”, which is involved in processing directional navigational information derived from the Earth´s magnetic field. Cluster N is medially joined by the hippocampal formation, known to retrieve and utilise navigational information. To investigate the connectivity and neurochemical characteristics of Cluster N and the hippocampal formation of migratory birds, we performed morphological and histochemical analyses based on the expression of calbindin, calretinin, parvalbumin, glutamate receptor type 1 and early growth response protein-1 in the night-migratory Garden warbler (*Sylvia borin*) and mapped their mutual connections using neuronal tract tracing. The resulting expression patterns revealed regionally restricted neurochemical features, which mapped well onto the hippocampal and hyperpallial substructures known from other avian species. Magnetic field-induced neuronal activation covered caudal parts of the hyperpallium and the medially adjacent hippocampal dorsomedial/dorsolateral subdivisions. Neuronal tract tracings revealed connections between Cluster N and the hippocampal formation with the vast majority originating from the densocellular hyperpallium, either directly or indirectly via the area corticoidea dorsolateralis. Our data indicate that the densocellular hyperpallium could represent a central relay for the transmission of magnetic compass information to the hippocampal formation where it might be integrated with other navigational cues in night-migratory songbirds.

## Introduction

**Fig. 1:**
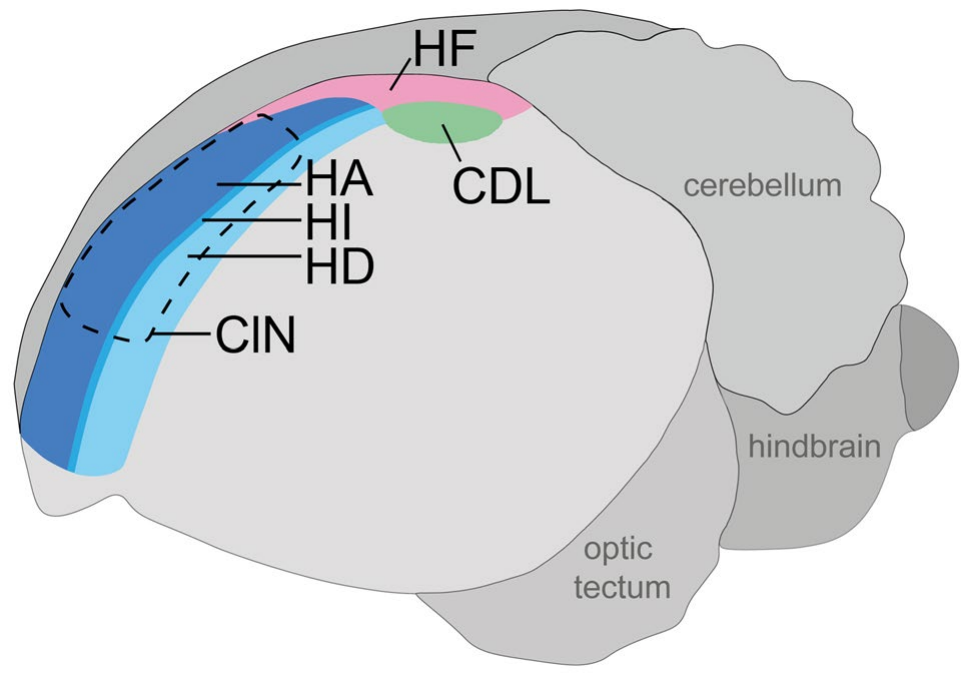
Schematic of the location of the hyperpallium and the hippocampal formation within the Garden warbler brain, sagittal view

The avian “Wulst” represents an elevated structure on the dorsomedial aspect of the forebrain which strongly differs in its extent between bird species. It is bounded laterally by the vallecula and can roughly be subdivided into a rostral (somatosensory) and a caudal (visual) subdivision. The Wulst consists of four hyperpallial laminae, i.e. the hyperpallium apicale (HA), intercalated part of hyperpallium apicale (IHA), hyperpallium intercalatum (HI) and hyperpallium densocellulare (HD) (Reiner et al. 2004; Jarvis et al. 2005; Fig. 1). The hyperpallium represents the major termination area for input from the thalamofugal visual pathway in birds (for review, see Güntürkün et al. 1993; Güntürkün 2000; Mouritsen et al. 2016). Its precise role in vision has long remained enigmatic, because earlier lesion studies revealed little to no effects on e.g. pattern, colour or intensity discrimination and visual acuity. Only later it was shown that the largest, posterior part comprises components of highly complex neuronal networks underlying various aspects of visual perception, e.g. contour discrimination (Nieder and Wagner 1999; Budzynski and Bingman 2004) and it was suggested to be involved in navigation- and orientation-related processes, such as sun compass associative learning (Budzinsky et al. 2002). More recently, a cluster of brain areas in the lateral posterior Wulst, “Cluster N” (Fig. 1), was repeatedly shown to display strongly increased neuronal activation in several night-migratory songbird species when performing magnetic compass orientation under low-light conditions (Mouritsen et al. 2005; Heyers et al. 2007; Liedvogel et al. 2007a; Hein et al. 2010; Zapka et al. 2009; 2010; Rastogi et al. 2011; Wu and Dickman 2011; Elbers et al. 2017). Activation of Cluster N is triggered by low-light vision and could not be observed in resident songbirds and/or species migrating during day (Mouritsen et al. 2005; Zapka et al. 2010). Based on these findings, Mouritsen et al. (2005) proposed that this night-vision processing could be related to a vision-mediated magnetic compass based on radical-pair-forming sensor molecules, cryptochromes, located in the birds eyes (Ritz et al. 2000; Hore and Mouritsen 2016; Mouritsen 2018, 2021). Cryptochromes are currently considered the most likely molecular basis for a radical pair-based magnetic compass sense in birds (Möller et al. 2004; Mouritsen et al. 2004b; Liedvogel et al. 2007b; Niessner et al. 2011, 2016; Bolte et al. 2016, 2021; Hore and Mouritsen 2016; Günther et al. 2018; Zoltowski et al. 2019; Einwich et al. 2020, 2021; Xu et al. 2021). Connectivity studies have shown that the eye and Cluster N are interconnected via parts of the thalamofugal visual pathway (Heyers et al. 2007). Currently, the suggestion that Cluster N is part of a neural circuit processing magnetic compass information is strongly supported by a study, which showed that, when Cluster N is chemically lesioned, night-migratory European Robins (*Erithacus rubecula*) are unable to use their magnetic compass, whereas their sun and star compasses are unaffected (Zapka et al. 2009).

At its caudal end, the Wulst is medially joined by the hippocampal formation (HF; Fig. 1), which can be roughly subdivided into a dorsolateral (DL), dorsomedial (DM) and a ventrally attached V-shaped region (Atoji and Wild 2004; Herold et al. 2014, 2019; Striedter 2016). Compelling evidence implicate the HF in spatial orientation, cognition and memory (Casini et al. 1997; Mayer et al. 2013; Herold et al. 2015; Sherry et al. 2017; Gagliardo et al. 2020): (1) variations in hippocampal morphology were shown to correlate with migratory behaviour (e.g. Krebs et al. 1989; Pravosudov et al. 2006; Bingman and MacDougall-Shackleton 2017); (2) HF subdivisions seem to display increased neuronal activation-triggered expression levels of immediate early genes in pigeons (*Columba livia*) when navigating by familiar landmarks within their home range (Shimizu et al. 2004), during the formation of spatial memory (Vargas et al. 2004) in a context-dependent manner in cowbirds (*Molothrus ater*) as they navigate through space (Grella et al. 2016), and in zebra finches (*Taeniopygia guttata*) when stimulated by magnetic fields (Keary and Bischof 2012); (3) single neuronal subpopulations in the pigeon’s HF were also reported to increase their firing rates following magnetic stimulation (Vargas et al. 2006); (4) recent evidence from Japanese quails (*Coturnix coturnix*) suggests the existence of avian analogues to mammalian head direction cells within HF (Ben Yishay et al. 2021); (5) place cells have been detected in the HF of tufted titmice (*Parus bicolor*), which are food-caching birds able to remember hundreds of locations for food caches (Sherry and Hoshooley 2007; Payne et al. 2021) as well as in pigeons (Hough and Bingman 2004; 2008); (6) recently, it has been shown that lesions of the HF of pigeons were found to disrupt a pigeon´s learned ability to discriminate magnetic intensity changes (Bingman et al. 2021).

Migratory birds will almost certainly have a particularly high need to integrate navigational information from various sensory sources to calculate their intended headings. However, until now, the morphological and biochemical characteristics and connectivity of Cluster N and the HF in migratory birds have remained elusive. In addition, Cluster N could only be characterized functionally based on behavioural molecular mapping techniques requiring time-consuming and carefully controlled behavioural experiments (Mouritsen et al. 2005; Heyers et al. 2007; Liedvogel et al. 2007a; Hein et al. 2010; Zapka et al. 2010; Rastogi et al. 2011; Wu and Dickman 2011; Elbers et al. 2017) based on the detection of immediate early genes, such as early growth response protein-1 (Egr-1, also known as ZENK), whose expression is driven by neuronal activation (Mello and Clayton 1995; Jarvis and Nottebohm 1997).

Thus, the aims of this study were to anatomically characterize both Cluster N and HF in a night-migrating songbird species using biochemical markers, which would then allow us to place both structures into the general avian neurochemical forebrain network. In addition, we aimed to specify potential connections from Cluster N with which magnetic compass information could reach HF for potential integration with other navigational cues. To do so, we mapped magnetic orientation-induced neuronal activation in the Wulst of Garden warblers (*Sylvia borin*), which represents the first species in which Cluster N was described (Mouritsen et al. 2005; Heyers et al. 2007; Hein et al. 2010), using an antibody against the immediate early gene Egr-1. The resulting expression pattern was mapped onto the expression patterns of selected members of the group of calcium binding proteins: calbindin (CB), calretinin (CR) and parvalbumin (PV), which have widely been used to characterize neuronal subpopulations/neuronal subcircuits in all parts of the nervous system throughout the vertebrate animal kingdom (Celio et al. 1986; Braun et al. 1996; Roberts et al. 2002; Guirado et al. 2003; Veney et al. 2003; Krützfeldt and Wild 2004, 2005; Wild et al. 2005; Suarez et al. 2005, 2006; Heyers et al. 2008; Logerot et al. 2011). These were complemented by additional expression analyses of glutamate receptor type-1 (GluR1; Wada et al. 2004) due to published findings of a previously unknown, GluR1-positive nucleus within Cluster N, the dorsal nucleus of the hyperpallium (DNH; Mouritsen et al. 2005; Zapka et al. 2010). Based on the resulting neurochemical “profile” of each of the investigated brain areas, we mapped the detailed neuronal connections within and between Cluster N and HF using neuronal tract tracing. We herein use the terminology introduced by Reiner et al. (2004). Based on genetic expression patterns, several studies have subsequently opted for a change of this nomenclature with respect to mesopallium and hyperpallium (Jarvis et al. 2013; Gedman et al. 2021). We go on using the terminology of Reiner et al. (2004) for reasons that will be outlined in the discussion.

## Materials and methods

### Animals and housing

Twelve Garden warblers (*Sylvia borin*) were obtained from bird stations in Helgoland (Germany), Rybachy (Russian federation) or were caught in the vicinity of the campus of Oldenburg University. Birds were housed in single wire cages (102 cm × 50 cm × 40 cm) under the natural circadian and circannual light conditions of Oldenburg. Food and water were provided ad libitum. All animal procedures were approved by the Animal Care and Use Committees of the Niedersächsisches Landesamt für Verbraucherschutz und Lebensmittelsicherheit (LAVES, Oldenburg, Germany, Az.: 33.42502/27–01.05; 33.19–42,502-04–15/1865; 33.19–42,502-04–20/3492) for the use of animals in research.

### Neuronal tract tracing

Birds were either anaesthetized by intramuscular injection of Ketamine (10%, 0.1 ml/kg body weight; Pharmanovo, Hannover, Germany)/Medetomidine (Domitor^©^, 0.1%, 0.1 ml/kg body weight; Orion Pharma, Espoo, Finland) or using Isoflurane CP^©^ 1–1.5% (1 ml/ ml; cp-pharma, Burgdorf, Germany) administered through a beak mask and were head-fixed in a custom-built stereotactic unit. The surfaces of both telencephalic hemispheres and the cerebellum were positioned in the same horizontal plane resulting in an angle of the plane between the tip of the beak and the ear bars of approximately 45º below the horizontal zero plane of the apparatus. The birds’ scalp was anaesthetized using a surface anaesthetic (Xylocain; Astra Zeneca, Wedel, Germany), incised, and temporarily pulled aside. Replicable tracer injections were achieved by using coordinates of the target structures relative to the confluence of the superior sagittal and cerebellar “Y” blood sinus providing the zero coordinate. 30–100 nl of 1% cholera toxin B subunit (CtB; Sigma, Deissenhofen, Germany) dissolved in phosphate buffered saline (PBS) was stereotactically applied using a microinjector (WPI Nanoliter 2000 Injector, Sarasota, FL, USA) through a small hole in the skull above the targeted brain region.

After the surgery, all incisions were closed and resealed with surgical glue (Histoacryl^®^, BRAUN, Rubi, Spain). In case of intramuscular anaesthesia, the effect of Medetomidine was antagonized using Atipamezole (Antisedan^©^, 0.5%, 0.1 ml/kg; Orion Pharma, Espoo, Finland). Birds were given at least 96 h to recover from the surgery and to let the tracer transport. Post-surgical analgesia was provided through intramuscular administration of Metacam^©^ (0.1 ml/kg body weight in 0.9% NaCl, Boehringer Ingelheim, Ingelheim, Germany) for 72 h.

### Behaviourally driven neuronal activation

Single birds were placed into a custom-built, cylindrical plexi-glass cage fitted with a circular perch (Mouritsen et al. 2004a, b, 2005; Heyers et al. 2010). To allow acclimatization to the new surroundings, birds were placed in the cage at least 30 min before the experiment started. At dusk, room lights were turned down to a light intensity of ~ 2.5 mW/m^2^, which equals strong moonlight and serves as a typically used value for countless behavioural orientation tests using night migrants (Wiltschko and Wiltschko 1972; Wiltschko et al. 1993; Mouritsen and Larsen 2001; Muheim et al. 2002; Mouritsen et al. 2005; Heyers et al. 2007, 2010; Liedvogel et al. 2007a; Hein et al. 2010, 2011; Zapka et al. 2009, 2010; Lefeldt et al. 2015; Elbers et al. 2017; Schwarze et al. 2016a, b; Kobylkov et al. 2019; Leberecht et al. 2022). Light was produced by incandescent light bulbs (wavelength spectrum given in Zapka et al. 2009). Each bird´s behaviour was continuously observed using infrared cameras (840 nm) connected to a surveillance monitor. Since any excess motor activity, such as flying around/jumping on/off the perch, would have led to motor-dependent activation in the brain (Feenders et al. 2008), birds were only collected after they had been sitting still but constantly awake for at least 90 min in order to keep brain activity evoked through any sensory or motoric disturbances as low as possible.

### Brain tissue processing

The birds were deeply anaesthetized with pentobarbital (Narcoren^®^, Boehringer Ingelheim, Ingelheim, Germany; 2.5 ml/kg body weight) under dim light conditions (~ 2,.mW/m^2^) and transcardially perfused with 0.9% saline containing 0.1% heparin sodium salt followed by 4% paraformaldehyde (PFA) dissolved in PBS. Brains were dissected, postfixed in 4% PFA overnight and cryoprotected in 30% D( +)-sucrose dissolved in PBS. Each brain was cut in six parallel series using a cryotome (Leica CM1860, Wetzlar, Germany) in sections of 40 µm thickness in either a frontal or sagittal plane. Until being subjected to immunohistochemical staining, sections were stored in PBS containing 0.1% sodium azide at 4 °C.

### Antibody characterization

The following primary antibodies were used in this study.

### Immunohistochemical stainings

Brain slices were stained free-floating using the immuno-ABC-technique (Heyers et al. 2007, 2008, 2010. Lefeldt et al. 2014; Elbers et al. 2017; Kobylkov et al. 2020; Haase et al. 2022). Each incubation step was followed by rinsing brain sections three times in PBS for 5 min each. Endogenous peroxidases were saturated by incubation in 0.3% hydrogen peroxide dissolved in distilled water for 30 min. Unspecific binding sites were blocked by incubating the slices in 10% foetal calf serum (Kraeber, Ellerbek, Germany) dissolved in PBS containing 0.3% Triton-X 100 (PBS-T) for 60 min. Slices were incubated with one of the primary antibodies overnight (3 days in case of Egr-1; Table 1) at 4ºC with gentle agitation. Afterwards, slices were sequentially incubated for 60 min each with biotinylated secondary antibodies and an avidin-coupled peroxidase-complex (Vector ABC Elite Kit, Vector Laboratories, Burlingame, CA, USA). Peroxidase-activity was detected using a 3′3-diaminobenzidine (DAB; Sigma, Deissenhofen, Germany) reaction, modified by using b-d-glucose/glucose-oxidase (Sigma, Deissenhofen, Germany) instead of hydrogen peroxide (Shu et al. 1988) to improve signal/background ratio. The substrate reaction was stopped in 0.1 M sodium-acetate. Slices were mounted on gelatinized glass slides, dehydrated in a graded series of ethanol (70%, 96%, isopropanol, xylene) and coverslipped with Entellan (Merck, Darmstadt, Germany).

**Table 1:** Primary antibodies used in this study

Name	Host	Dilution (Immuno-histo-chemistry)	Company	ID	RRID
Calbindin (CB)	Rabbit	1:1000	Swant	CB38a	AB_10000340
Calretinin (CR)	Rabbit	1:1000	Swant	7699/4	AB_10000321
Cholera Toxin B subunit (CtB)	Rabbit	1:1000	Sigma	C3062	AB_258833
Early growth factor 1 (EGR1)	Rabbit	1:1000	Santa Cruz	sc-189	AB_2231020
Glutamate receptor 1 (GluR1)	Rabbit	1:500	Chemicon	AB1504	AB_90705
Parvalbumin (PV)	Mouse	1:500	Sigma	P3088	AB_22592925

### Imaging and analysis

Slides were imaged with a light microscopic slide scanner (Zeiss Axio Scan.Z1, Oberkochen, Germany). Images used in this article were equally adjusted in contrast/brightness using ImageJ (NIH, Bethesda, MD, USA; Schindelin et al. 2012). Schematic drawings, labelling and layout were made using a pen display (Wacom Intuos Pro, Krefeld, Germany) and Photoshop / Illustrator software (Adobe Systems, Mountain View, CA, USA). Since no brain atlas for the Garden warbler is available to date, the neuroanatomical analyses were performed using the brain atlases of chicken (Kuenzel and Masson 1988; Puelles et al. 2007), pigeon (Karten and Hodos 1973), canary (Stokes et al. 1974) and zebra finch (Nixdorf-Bergweiler and Bischof 2007; Lovell et al. 2020).

## Results

**Fig. 2:**
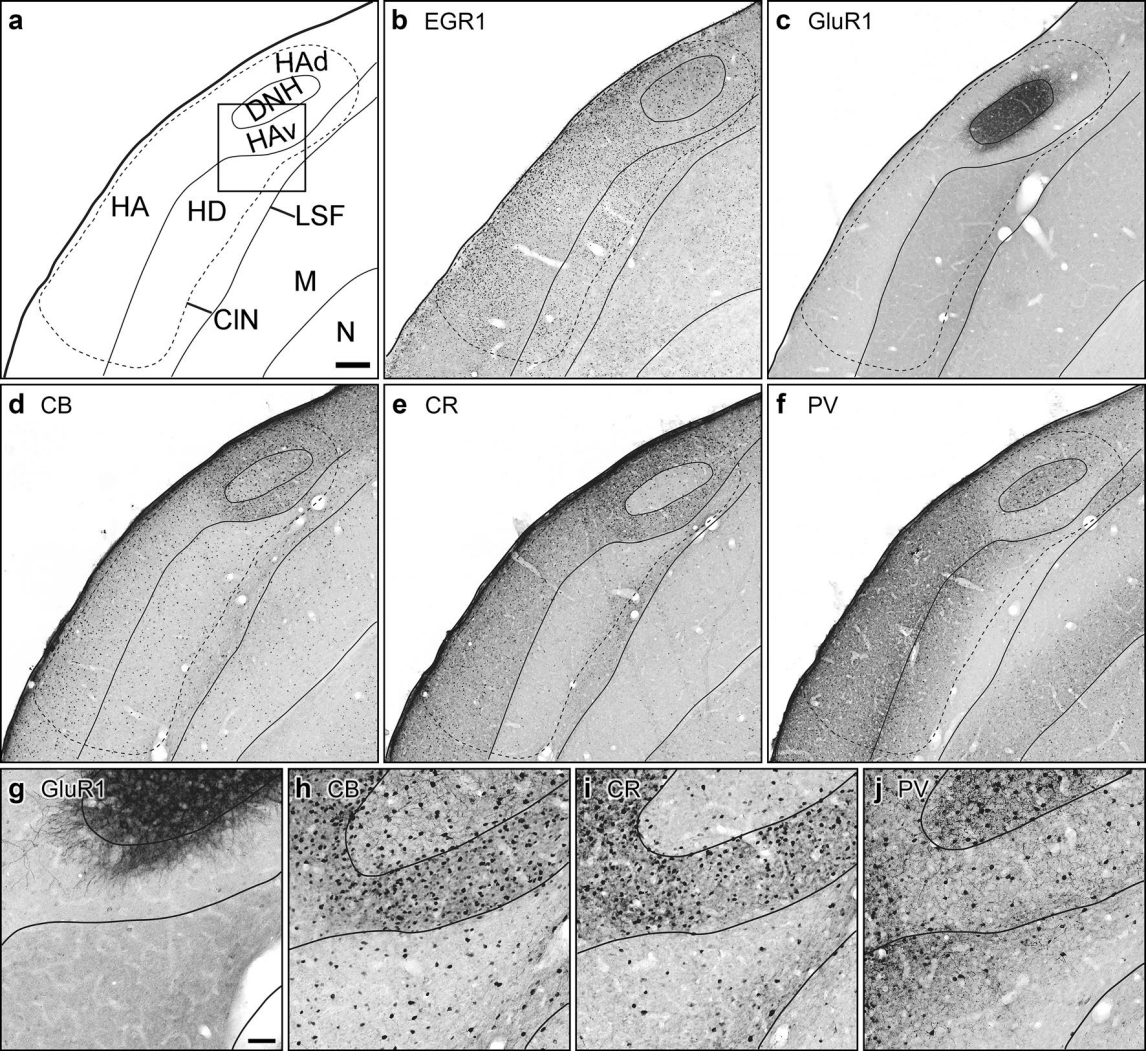
Anatomy and biochemistry of the Garden Warbler Wulst, sagittal view. **a** Schematic drawing including subdivisional boundaries for anatomical orientation. Insert shows the location of magnified details displayed in **g**, **h**, **i** and **j**. Parallel sagittal sections at the level of DNH (L ~ 2.5) immunohistochemically processed against Egr-1 (**b**), GluR1 (**c**), Calbindin (CB; **d**), Calretinin (CR; **e**) and Parvalbumin (PV; **f**). Note the magnetoreception-triggered dim-light activation, as depicted by Egr-1-expressing nuclei **b**. Scale bar = 200 µm in (**a**), for (**a**)-(**f**). Magnified details of the respective marker staining: (**g**), GluR1, (**h**), CB, (**i**), CR, (**j**), PV. Scale bar in (**g**) (for (**g**)-(**j**)) = 50 µm. For abbreviations, see list

**Fig. 3:**
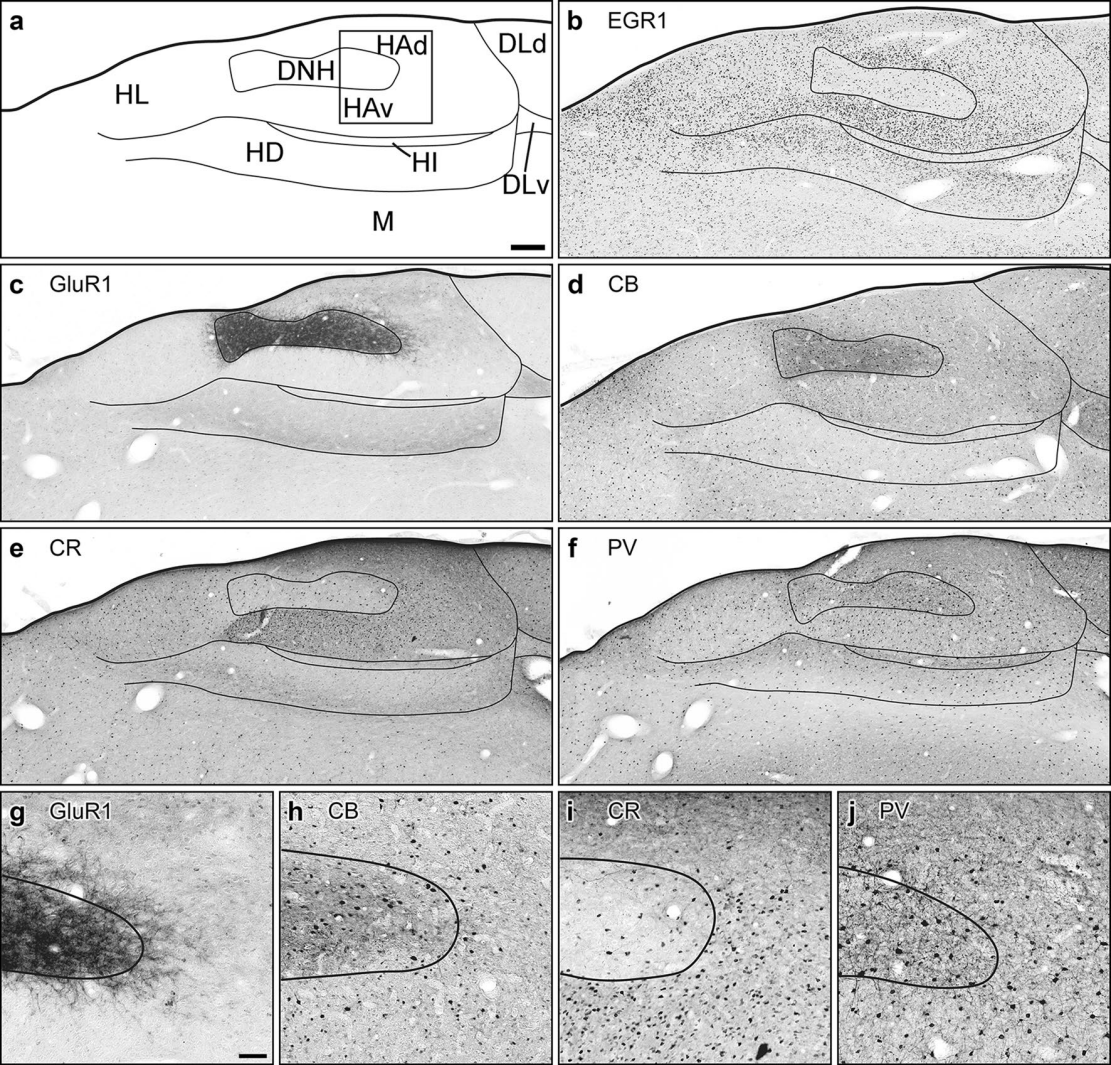
Anatomy and biochemistry of the Garden Warbler Wulst, frontal view. **a** Schematic drawing including subdivisional boundaries for anatomical orientation. Insert shows the location of magnified details displayed in **g**, **h**, **i** and **j**. Parallel frontal sections at the level of Cluster N immunohistochemically processed against Egr-1 (**b**), GluR1 (**c**), Calbindin (CB; **d**), Calretinin (CR; **e**) and Parvalbumin (PV; **f**). Note the magnetoreception-triggered dim-light activation, as depicted by Egr-1-expressing nuclei **b**. Scale bar = 400 µm in (**a**), for (**a**)-(**f**). Magnified details of the respective marker staining: (**g**), GluR1, (**h**), CB, (**i**), CR, (**j**), PV. Scale bar in (**g**) (for (**g**)-(**j**) = 150 µm. For abbreviations, see list

**Fig. 4:**
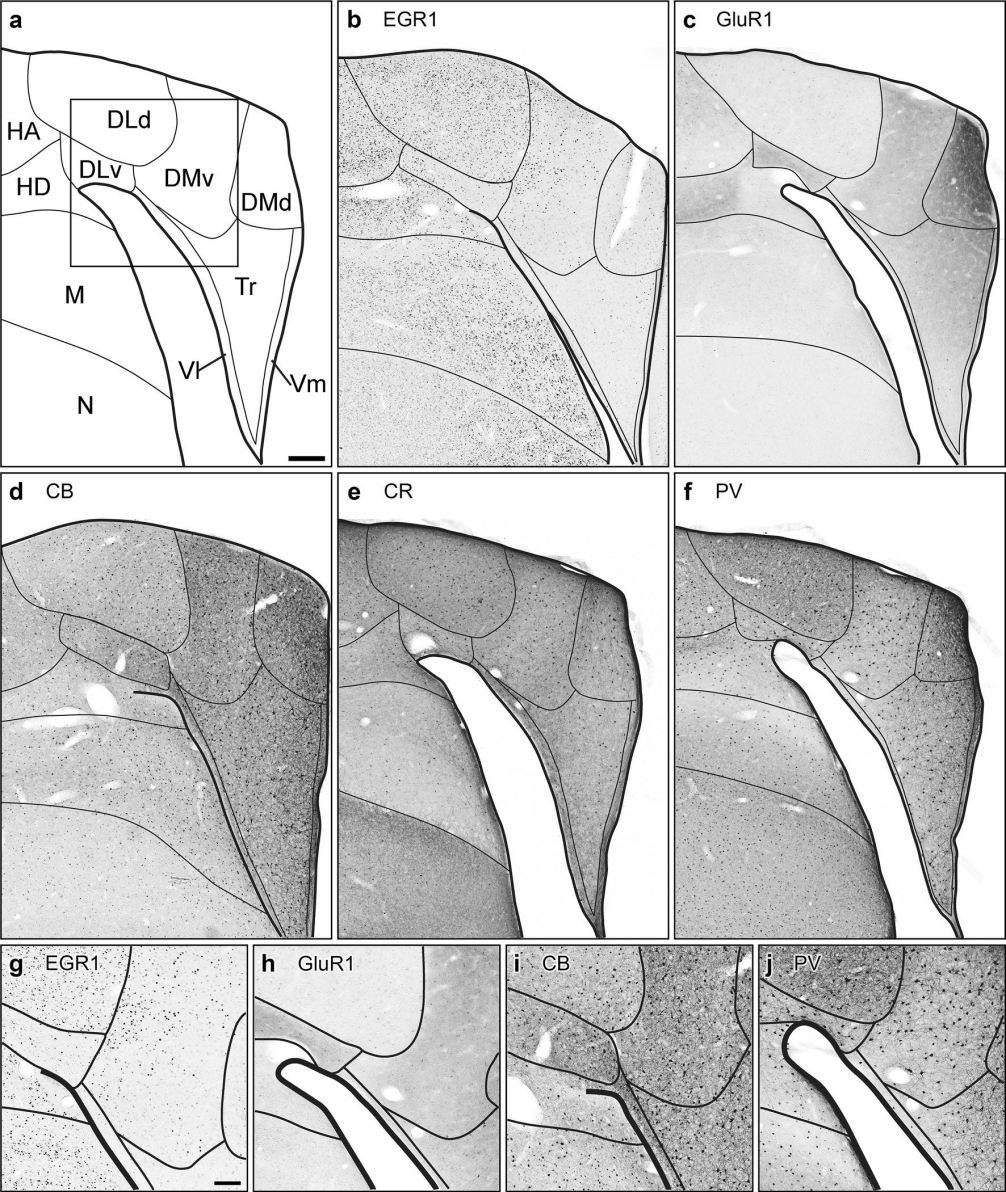
Anatomy and biochemistry of the Garden Warbler hippocampal formation, frontal view. (**a**) Schematic drawing including subdivisional boundaries for anatomical orientation. Insert shows the location of magnified details displayed in **g**, **h**, **i** and **j**. Parallel frontal sections at the level of Cluster N immunohistochemically processed against Egr-1 (**b**), GluR1 (c), Calbindin (CB; d), Calretinin (CR; e) and Parvalbumin (PV; f). Note the magnetoreception-triggered dim-light activation, as depicted by Egr-1-expressing nuclei **b**. Scale bar = 200 µm in (a), for (a)-(f). Magnified details of the respective marker staining: (**g**), Egr-1, (**h**), GluR1, (**i**), CB, (**j**), PV. Scale bar in (**g**) (for (**g**)-(**j**)) = 50 µm. For abbreviations, see list

**Fig. 5:**
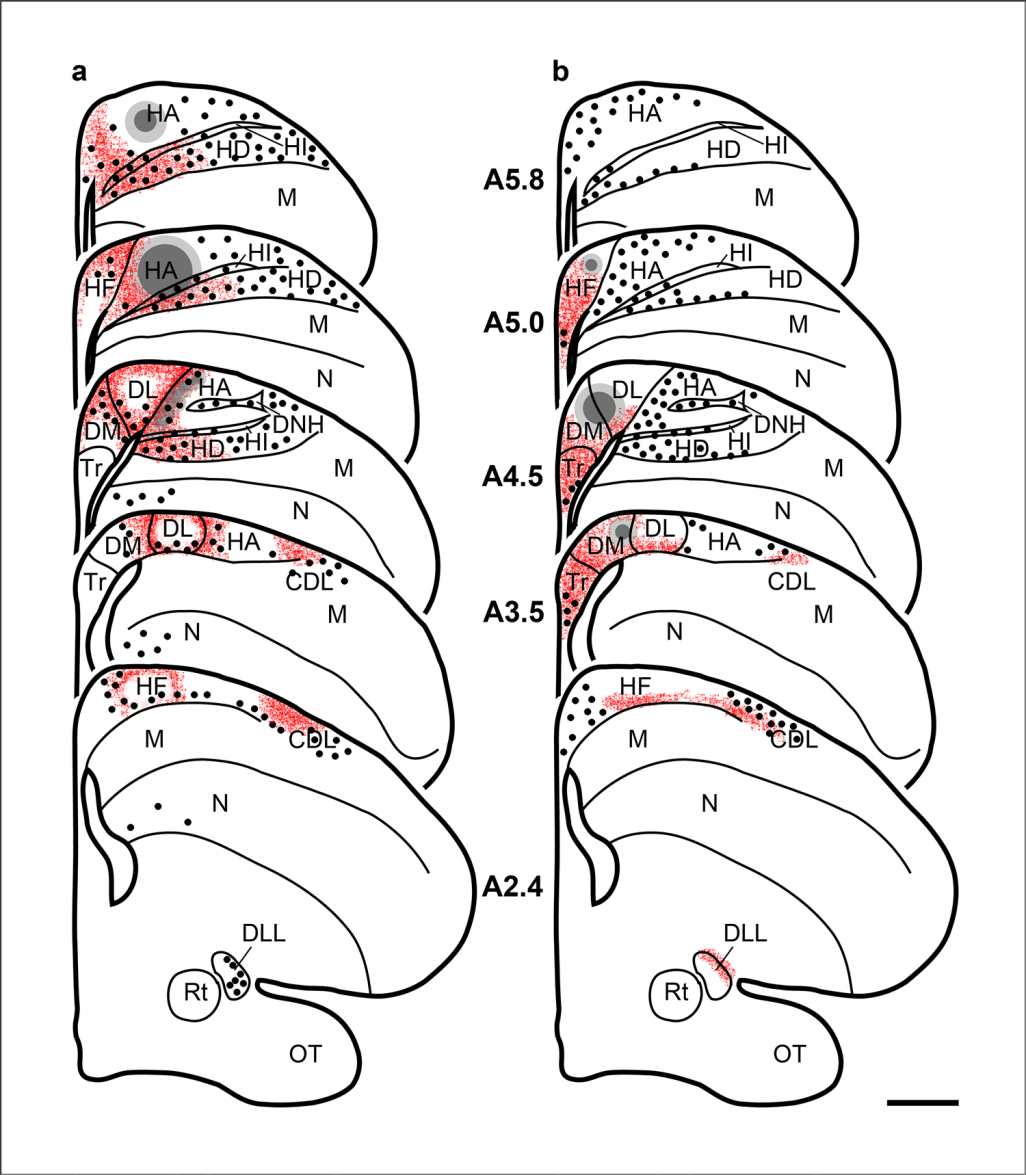
Schematic illustration of the rostrocaudal extent of labelling following tracer injections into Cluster N **a** and HF **b**. Location of core injection is marked in dark grey, while the grey area around the core indicates tracer spread. For illustration purposes, retrogradely labelled neurons are represented by black dots, while the locations of anterogradely labelled fibres and terminals are marked in red. Neither the dots nor the red signal represent real numbers. Scale bar = 1 mm. For abbreviations, see list

**Fig. 6:**
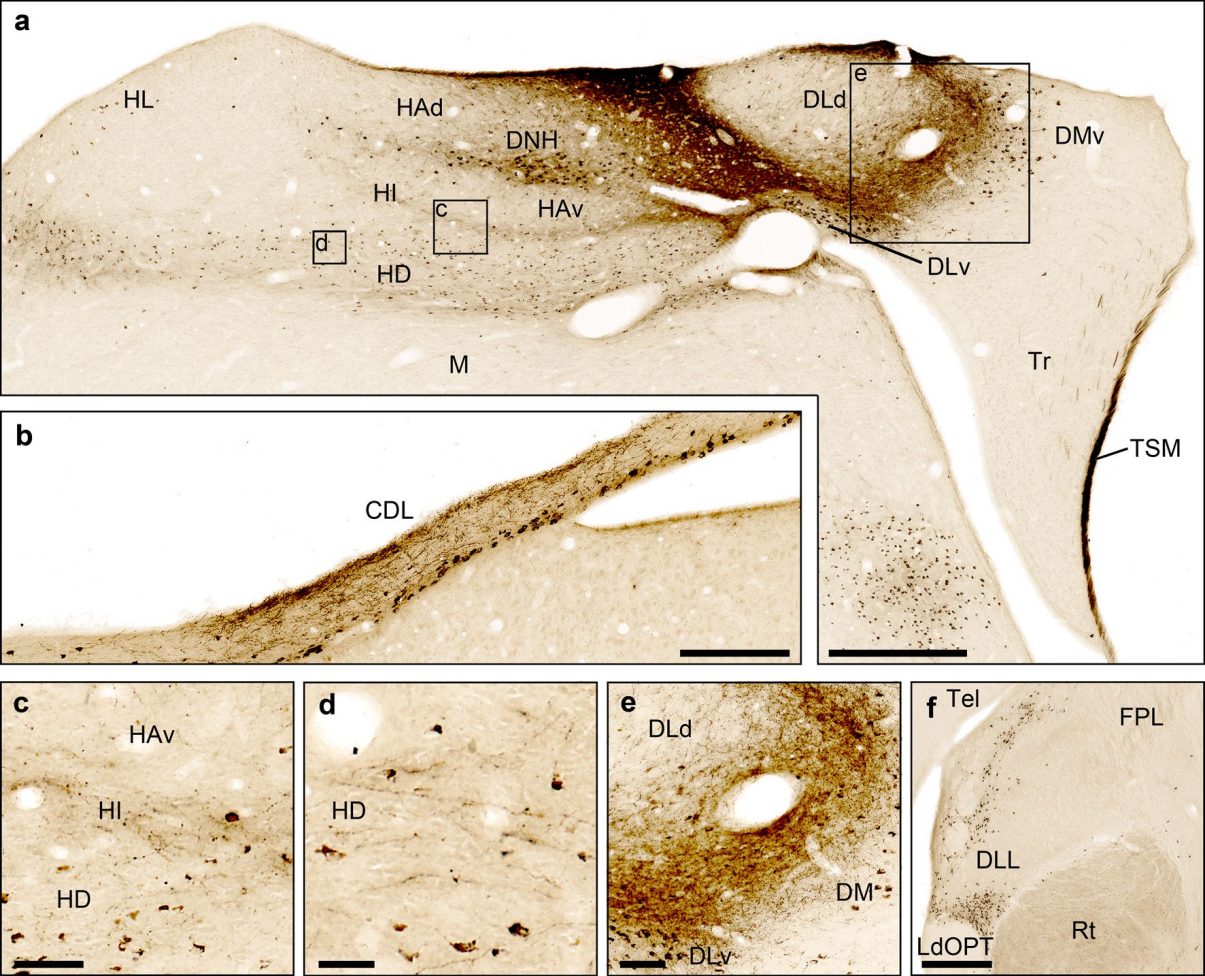
Connectivity of the Garden Warbler Wulst, frontal view. (**a**) Neuronal tract tracing pattern within the Wulst after injection of Cholera toxin B subunit into the centre of Cluster N. Inserts show the location of magnified details displayed in **c**, **d** and **e**. Scale bar = 500 µm. (**b**) Resulting tracing patten in the dorsolateral corticoid area (CDL) approximately 500 µm posterior to the injection site. Note the dense pattern of anterogradely labelled fibre terminals in dorsal and retrogradely labelled somata in ventral CDL parts. Scale bar = 25 µm. (**c**) Anterogradely labelled fibre terminals in medial HI parts. Scale bar = 50 µm. (**d**) HD receives both anterograde and retrograde input after tracer injection into Cluster N. Scale bar = 25 µm. (**e**) Anterogradely labelled fibre terminals at the hippocampal DM/DL transition zone. Retrogradely labelled neurons are found in DLv and DMv compartments. Scale bar = 100 µm. (**f**) Retrogradely labelled neurons in lateral and ventral compartments of the ipsilateral GLd after tracer injection into Cluster N. Scale bar = 100 µm. For abbreviations, see list

**Fig. 7:**
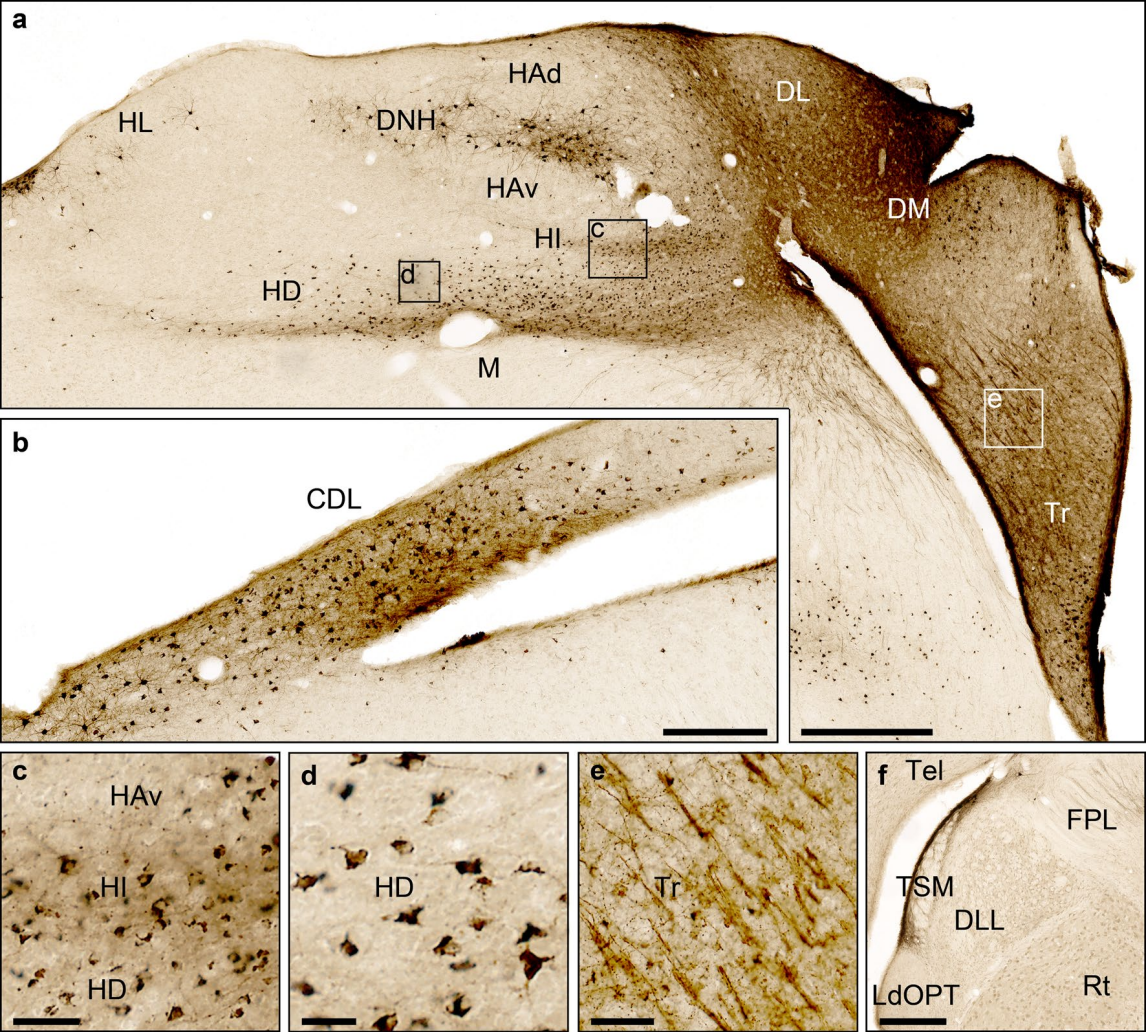
Connectivity of the Garden Warbler hippocampal formation, frontal view. **a** Neuronal tract tracing pattern within the Wulst after injection of Cholera toxin B subunit into DM/DL. Inserts show location of magnified details displayed in **c**, **d** and **e**. Scale bar = 500 µm. **b** Resulting tracing patten in the dorsolateral corticoid area approximately 500 µm posterior to the injection site. Note the dense pattern of anterogradely labelled fibre terminals in dorsal and retrogradely labelled somata in ventral CDL parts. Scale bar = 25 µm. (**c**) Dense retrograde labelling of neurons in medial Cluster N parts HA, HI and HD after hippocampal tracer injections. Scale bar = 50 µm. **d** Strong retrograde tracer transport from the hippocampal formation to HD. Scale bar = 30 µm. **e** Tracer injections into DM/DL reveal dense anterograde innervation in the triangular hippocampal subdivision (Tr). Scale bar = 100 µm. **f** Anterogradely labelled fibres within the septomesencephalic tract (TSM) of the ipsilateral thalamic GLd roof. Scale bar = 100 µm. For abbreviations, see list

We assessed the general morphological and biochemical properties of the hyperpallium (containing Cluster N) and the HF using immunohistochemical staining against Egr-1, GluR1, CB, CR and PV on parallel slice series of Garden warbler forebrains. Patterns of immunohistochemical staining are displayed on representative brain slices at the level of Cluster N cut in a sagittal (Fig. 2) and frontal plane (Figs. 3, 4). Each figure contains schematic inserts for anatomical orientation (Figs. 2a, 3a, 4a). Neuronal connectivity to/from Cluster N and HF was visualized using the neuronal tract tracer Cholera toxin B subunit injected into Cluster N and HF (Figs. 5, 6, 7).

### Egr-1

Low-light-induced neuronal activation of Cluster N, depicted by the expression of the immediate early gene Egr-1, was observed in a brain area spanning ~ 1 mm in the rostrocaudal, ~ 2 mm in the dorsoventral and ~ 2.5 mm in the mediolateral axis. This area was located at the posterior pole of the hyperpallium and, in sagittal sections, was dorsally bordered by the HF (Fig. 2b). In the hyperpallium, Egr-1 expression covered all major hyperpallial compartments with highest densities in ventral HA (HAv) parts (formerly described as “DNH shell”; Mouritsen et al. 2005) and HD (Fig. 3b). HD displayed a gradient of Egr-1 positive neurons with declining numbers towards the mesopallial border. DNH, as previously shown (Mouritsen et al. 2005; Zapka et al. 2010; Elbers et al. 2017), was found largely devoid of Egr-1 (Fig. 3b). In HF, high Egr-1 expression was observed in the hippocampal DLd and adjacent lateral DMv subdivisions, whereas the remaining DLv, DMd and the V-shaped region of the HF exhibited little to no Egr-1 expression (Fig. 4b, g).

### GluR1

Apart from few dispersed GluR1-positive neurons in HAd, the DNH represented the most prominent hyperpallial brain structure characterized by a dense, GluR1-positive fibre network (Figs. 2c, g, 3c, g), while the surrounding HAv and HL were largely devoid of GluR1 Fig. 2c, g; Fig. 3c, g). HD was characterized by diffuse GluR1 expression, which extended into the underlying mesopallium (Fig. 2c; 3c). In HF, GluR1 prominently labelled DMd, while DMv, DLv and the V-shaped region were moderately labelled. DLd was entirely devoid of GluR1, thus setting a conspicuous boundary between the hyperpallium and the medially adjacent HF (Fig. 4c, h).

### Calbindin

The Garden Warbler hyperpallium displayed increased numbers of CB expressing neurons at caudal HAd and HAv levels surrounding DNH (Fig. 2d, h), while CB immunosignal in HL and HD appeared low. DNH contained moderate numbers of CB positive cells (Figs. 2d, h, 3d, h). Sagittal sections revealed a slightly increasing CB expression gradient towards the frontal superior lamina (LFS), separating the hyperpallium from the underlying mesopallium (Fig. 2d). In HF, higher CB expression levels covered all major subcompartments with highest expression levels in DM, Vm and Vl, and thereby labelled a conspicuous boundary towards the only moderately labelled DL (Fig. 4d, i).

### Calretinin

CR expression in sagittal sections of the Garden Warbler hyperpallium homogenously covered the entire HA from its rostral to caudal extent (Fig. 2e, i). CR positive cells were most abundant in medial HAv (Fig. 3e, i). In HL and DNH, CR displayed low to moderate expression levels (Figs. 2e, i, 3e, i). In HD, we observed a slightly increasing dorsal to ventral expression gradient, which peaked in LFS (Figs. 2e, 3e). In HF, CR expression was less distinct but appeared to label its subdivisional borders (Fig. 4e).

### Parvalbumin

In sagittal sections, PV displayed a uniformly high expression pattern in superficial layers of the rostral hyperpallium (Fig. 2f, j), thereby showing an almost complementary expression pattern as compared to CB (Fig. 2d, h). In HD, PV followed a gradually decreasing expression level towards LFS (Figs. 2f, 3f). Around DNH, increased numbers of parvalbumin expressing neurons were found in HAv (Fig. 2f, j), thereby displaying a striking contrast to the DNH, which contained many PV-positive multipolar neurons with large dendritic fields (Figs. 2f, j, 3f, j). HAd was almost devoid of PV positive cells and only contained diffusely stained PV-positive fibres (Figs. 2j, 3j). In HF, strongest PV-immunoreactivity was observed in DMd and DLd, whereas the remaining DMv, DLv and V-shaped region only contained large, more dispersed occurring PV-expressing neurons.

### Neuronal tract tracing

Neuronal connectivity to/from Cluster N were visualized using focal injections of cholera toxin B subunit into Cluster N (*n* = 4; coordinates: A 5.0 mm; L3.0 mm; depths 0.5 mm; Fig. 5a, 6a) and HF (*n* = 4; coordinates: A 4.7 mm; L1.5 mm; depths 0.5 mm; Fig. 5b, 7a). In the following, we primarily focused on the connectivity within and between the hyperpallium, HF and the dorsolateral corticoid area (CDL).

To label as many Cluster N connections as possible, we administered considerably large volumes of neuronal tracer to intermediate aspects of the caudal Garden Warbler hyperpallium. We confirmed the correct tracer placement into the thalamorecipient layers of Cluster N by observing retrogradely labelled neurons in ventral parts of the thalamic GLd, which largely mirrored previously published data (Figs. 5a, 6f; Heyers et al. 2007). Within the hyperpallium, we observed a prominent band of retrogradely labelled neurons, which covered HD almost in its entirety (Figs. 5a, 6a, d). Retrogradely labelled neurons were further observed within HL and the DNH (Figs. 5a, 6a). Anterogradely traced fibres stretched throughout HD with highest fibre densities in medial parts (Figs. 5a, 6d). In addition, the tracer labelled a thin band of anterogradely labelled fibres ventrally adjacent to HA, potentially representing HI (Figs. 5a, 6a, c).

Within HF, we observed a dense network of anterogradely labelled terminals in DL and DMv. Fewer retrogradely labelled neurons were observed in the DMv and DLv subdivisions (Figs. 5a, 6a, e). The remaining HF subcompartments were largely devoid of any projections (Figs. 5a, 6a). A conspicuous bundle of thick fibres passed through DL to form the septopallio-mesencephalic tract (TSM). TSM took its course along the median sagittal edge of the hemisphere, dived caudoventrally to terminate in the thalamic GLd roof (Figs. 5a, 6a, f).

Outside HF and Cluster N, at more caudal levels, we observed a prominent anterograde projection to CDL approximately located at the lateral edge of the ventricle. A thin band of retrogradely labelled fibres were located in ventral CDL parts adjacent to the ventricle (Figs. 5a, 6b).

To confirm these results, we placed a tracer injection in DM/DL within HF, which had received anterograde projections from Cluster N. Prominent intrahippocampal anterograde projections covered all subdivisions of the V-shaped region, but most of them terminated in Tr (Figs. 5b, 7a, e). In addition, anterograde tracing was also observed in more caudal DLv parts.

Projections to and from the hyperpallium labelled by HF tracings were as follows: like in the hyperpallial tracings, we observed a prominent band of retrogradely labelled neurons in HD with the majority restricted to medial parts (Figs. 5b, 7a, c, d). Furthermore, retrogradely labelled neurons were found in medial HI and HA (Figs. 5b, 7a, c). In HA, the labelled neurons were located further rostrally within superficial HAd layers and, more sparsely distributed, in medial parts adjacent to DL. Prominently large retrogradely labelled multipolar neurons were located in DNH. Anterograde tracing was mainly observed within ventral aspects of HD (Figs. 5b, 7a).

Outside the HF and Cluster N, HF tracings revealed a strongly labelled patch of retrogradely labelled neurons in superficial CDL parts and a dense anterogradely labelled patch of fibres ventrally attached, thereby showing almost opposite connectivity as compared to Cluster N tracings (Figs. 5, 6b, 7b). At thalamic levels, we observed anterogradely labelled TSM fibre terminals within the GLd roof (Figs. 5b, 7f).

## Discussion

### General morphological/biochemical observations

Using immunohistochemical staining against GluR1, CB, CR and PV, we show that the subdivisions of the Garden Warbler hyperpallium match well with the subdivisional schemes known from other birds (Reiner et al. 2004; Jarvis et al. 2005). Each of them displays a unique biochemical profile: HA is subdivided into an anterior, strongly PV-positive, and a posterior, strongly CB-positive part, which forms a shell-like structure surrounding DNH, and whose expression extends into the caudally attached HF (Figs. 2d, f; 3d, f; 4d, f). CR expression homogenously covers the entire HA (but not DNH) and thus differs from all other proteins analyzed (Figs. 2e, 3e). DNH spans along intermediate depths within HA, shows immunoreactivity against ionotropic GluR1 receptor (Fig. 3c) and PV (Fig. 3f), and, additionally, is clearly distinguishable from the surrounding HA with all other CaBPs analyzed (Fig. 3). Finally, HD is characterized by PV expression (Figs. 2f, 3f), which displays a gradually decreasing gradient towards LFS, and CB (Fig. 3d) and CR (Fig. 3e), both of display an almost complementary expression pattern as compared to PV (Fig. 3f), i.e. with strongest expression levels in LFS.

Likewise, our immunostainings compartmentalize the Garden Warbler HF into its structural subdivisions known from pigeons (Herold et al. 2014): a DL portion, which is subdivided into a PV-positive DLd (Fig. 4f) and GluR1-positive DLv (Fig. 4c); DM displays strong PV (Fig. 4f) and GluR1 (Fig. 4c) expression in DMd, while DMv is characterized by CB (Fig. 4d) and a moderately strong expression of GluR1 (Fig. 4c). Most prominent expression feature of the V-shaped region are strongly CB (Fig. 4d) and CR-positive (Fig. 4e) fibres in Vl and Vm laterally flanking Tr.

### Hippocampal formation

The role of the avian HF in the context of navigation and spatial memory has long been studied (Herold et al. 2015; Mouritsen et al. 2016). In stark contrast, information is sparse when it comes to correlate hippocampal functions to specific subdivisions and even less when it comes to the role of the HF in using, modulating or integrating magnetic information. Here, only a few studies reported magnetic field-responsive neurons (Vargas et al. 2006; Wu and Dickmann 2011, 2012; Keary and Bischof 2012). How is this evidence connected to migratory behaviour and navigation and what are the neuronal correlates? Indeed, 20 years after the first reports of spatially active and location-sensitive neurons in the HF of pigeons (Siegel et al. 2002, 2005; Hough and Bingman 2004), studies reported place cells organized along the anterior–posterior axis of the HF in tufted titmice (food-hoarding songbird) and zebra finches (non-food hoarding songbird), as well as head direction cells in the HF of Japanese quails (Payne et al. 2021; Ben-Yishay et al. 2021). Furthermore, it was reported that hippocampal lesions disrupt the capacity of discriminating magnetic intensity but not inclination in pigeons, while hyperpallial lesions had the opposite effect, suggesting a double-dissociation of the HF and the hyperpallium (Bingman et al. 2021). However, no details of subdivisional restrictions in the last three mentioned studies were provided, although a common subdivisional scheme based on different morphology, neurogenic and neurochemical markers and connectivity patterns exists (Atoji and Wild 2006; Herold et al. 2014, 2019; Striedter 2016). This subdivisional scheme, as shown in the data presented here, also applies for the Garden warbler. Thereby, the observed differential distribution patterns of CaBPs (PV, CR, CB) and GluR1 allowed us to create a precise subdivisional scheme for the HF and additionally to separate the HF from the adjacent CDL and HA. The different expression patterns of CaBPs further suggest functional specializations of the subdivisions as to the known different contribution to neuronal excitability of selective CaBPs (Markram et al. 2004). In addition, our data show that during night-migratory behaviour, neurons in DLd and DMv of the Garden warbler, were highly activated, while all other subdivisions showed little to no Egr-1 activation. This shows for the first time that the HF is actively involved during night-migratory behaviour and will be discussed in more detail below together with the observed connectivity loop between the HF, HD and CDL and a possible involvement in the transmission and integration of magnetic information.

### Cluster N

High neuronal activation in the visual forebrain region Cluster N of migratory birds has been associated with processing of compass information during magnetic orientation (Mouritsen et al. 2005, Heyers et al. 2007; Liedvogel et al. 2007a; Hein et al. 2010; Zapka et al. 2009, 2010; Mouritsen et al. 2016; Elbers et al. 2017; Fig. 1). Night-migratory songbirds with lesioned Cluster N were unable to use their magnetic compass, whereas they could use their star and sun compasses (Zapka et al. 2009). Mouritsen et al. (2005) originally assigned the borders of Cluster N to all major hyperpallial layers and the ventrally adjacent dorsal mesopallium (MD). According to Jarvis et al. (2013), HD as the ventral most hyperpallial subdivision is of mesopallial origin based on the distribution of histogenetic markers. This anatomical designation of Jarvis et al. (2013) contradicts the existing anatomical nomenclature (Reiner et al. 2004; Shimizu et al. 1995) and recent tracing studies (Stacho et al. 2020). These demonstrated a dense and column-like interconnectivity between all four hyperpallial areas incl. HD while connections to MD were sparser. We, therefore, used in our study the official terminology, which is further supported by earlier neuronal tract tracing data, where the lateral portion of HD (MD sensu Jarvis et al. 2013) but not MD has been shown to receive visual input from the dorsal lateral thalamic geniculate complex (Karten et al. 1973).

Having the here presented morphological markers available, Cluster N can be assigned to the posterior parts of all major hyperpallial compartments (Fig. 2). In addition, we observed activated neurons in the medially adjacent hippocampal DLd and a thin band within DMv at the DM/DL border (Fig. 4b).

Although, admittedly, none of the analyzed markers selectively labelled Cluster N as a whole, the combinatorial expression patterns of the analyzed proteins mark the approximate borders of Cluster N (except for its rostral boundary towards the somatosensory Wulst part; Wild 1987, 1997) and thus allow us to place it into the known avian forebrain network. Moreover, our marker analyses further subdivide the Garden Warbler Wulst into biochemically distinct compartments, some of which could potentially be dedicated to a specific functional subsystem within the thalamofugal visual system.

### Wulst connectivities

In vivo neuronal tract tracings intrinsically bear two problems: (1) the accidental co-labelling of passaging fibres, particularly when mapping layered structures such as the bird forebrain; (2) depending on the size of the area of interest, precise injections are difficult to confine to the respective target. Brain slice cultures can at least partially circumvent those problems because target areas can be visually approached without having to penetrate neighbouring structures. In turn, any connectivity far away from the injection site will be cut off, while in vivo tracings potentially reveal labelled fibres and somata all over the brain.

We vindicated the use of in vivo tracing and used considerable amounts of neuronal tracer in our injections for two reasons: (1) Cluster N was previously considered as a functional entity and we wanted to label as many as possible potential connections; (2) the estimated number of experimental animals needed to perform precise focal tracings on a subdivisional level in the brain of a non-breedable, wild-caught, night-migratory songbird species, such as the Garden Warbler, would by far have exceeded both our legal and ethical limits. In order to nevertheless critically assess our own data, in the following, we took greatest care to evaluate the shown connectivity patterns by superimposing them onto the Wulst connectivity known from other bird species (e.g. Shanahan et al. 2013).

The hyperpallium in birds is a four-layered structure, consisting of HA, IHA, HI and HD. Its role as the main termination area for visual information from the thalamic GLd (Güntürkün, 1993, 2000) was first shown in a fibre degeneration study in pigeons, which showed that visual information is primarily projected onto IHA with weaker terminations in HD (Karten et al. 1973; Wild 1997). Later, retrograde tracings from the visual Wulst including HA confirmed the GLd as afferent thalamic source (Bagnoli and Burkhalter 1983; Miceli and Repérant 1985; Deng and Rogers 1998; Miceli et al. 1990; Güntürkün and Karten 1991; Ströckens et al. 2013). More recently, Atoji et al. (2018) showed additional thalamic projections to HI and HD in pigeons. Thus, GLd appears to originate visual information to all major layers of the hyperpallium to different extents. Our Cluster N tracings retrogradely labelled GLd neurons (Fig. 4f), which proves that thalamorecipient hyperpallial layers (i.e. HA, IHA, HI and/ or HD) have been successfully targeted.

Within the Wulst, IHA projects to HA and IH (Wild 1987; Kröner and Güntürkün, 1999; Shimizu et al. 1995; Stacho et al. 2020). As shown by Stacho et al (2020) HI and HA project to HD in pigeons. Furthermore, Nakamori et al. (2010) found a pathway involving three relays within HD.

**Fig. 8:**
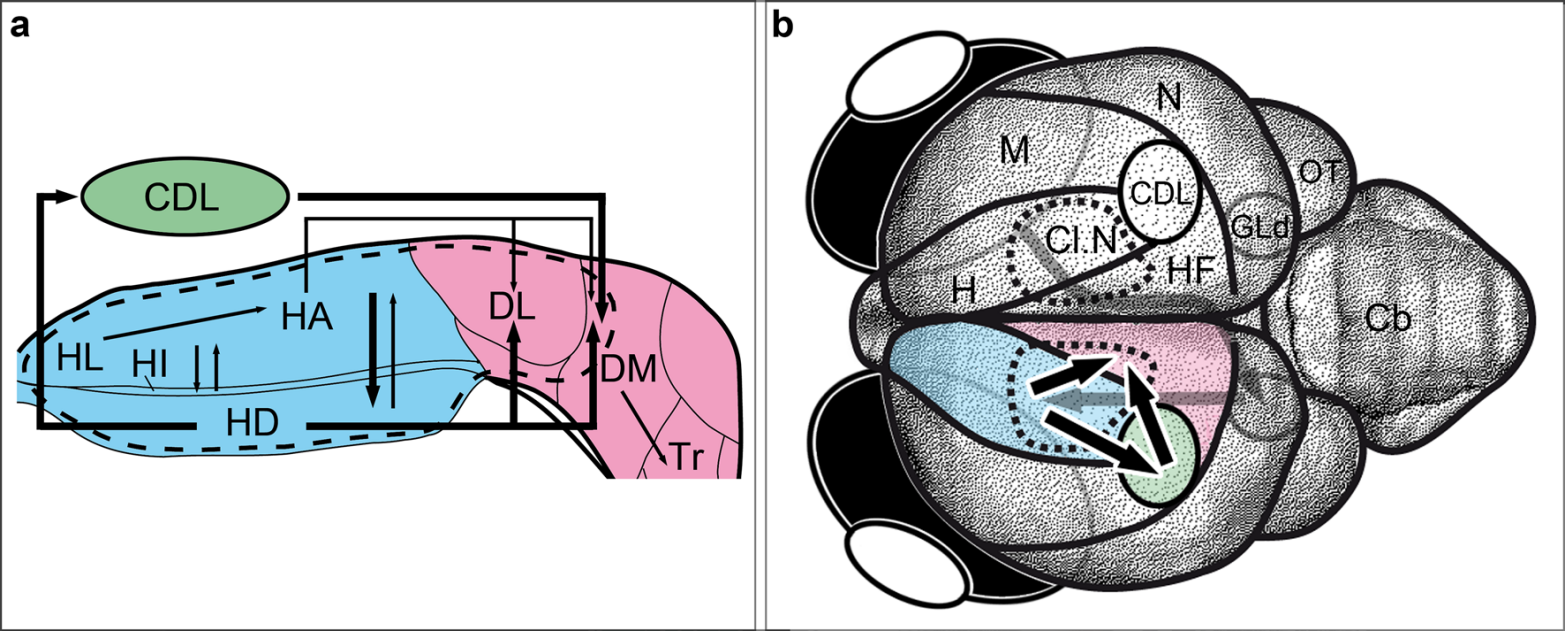
The proposed neural connectivity between Cluster N and the hippocampal formation in the Garden warbler. (**a**) Proposed connections based on the tracing results shown in Figs. 4 and 5. Boldness of arrows indicates strength of connections between the brain substructures. Note HD as the main origin of fibres reaching HF either directly or indirectly via CDL. (**b**) Global view of the proposed neural pathway underlying magnetic compass information processing (drawn unilaterally only for illustration purposes). Areas inside the dotted line display night vision-triggered activation (i.e. Cluster N and parts of the medially attached hippocampal formation). Blue = visual Wulst; Red = hippocampal formation; Green = CDL. For abbreviations, see list

Due to the coarseness of our very limited number of injections, not all of the above mentioned intrahyperpallial connectivity could be clearly revealed in our study. However, our Cluster N tracings confirmed massive reciprocal tracer transport to and from HD, and, to a lesser degree, HI, which indicates that our injection site included HA (Figs. 5a, 6a, c, d; 8; Shimizu et al. 1995; Kröner and Güntürkün, 1999).

Regarding hyperpallial-hippocampal connectivity, HA and HD in pigeons were shown to have reciprocal connections with DM (Casini et al. 1986; Kröner and Güntürkün, 1999; Atoji and Wild 2004)–an anatomical finding that was also substantiated by a resting-state connectivity analysis (Behroozi et al. 2017). In the Garden Warbler, we observed anterograde projections from Cluster N to DM and DL (Figs. 5a, 6a, e, 8), confirmed by retrograde tracings from HF, which retrogradely labelled many neurons in HD and, to a lesser degree, in HA (Figs. 5b; 7a; 8). In addition, HD in pigeons was previously shown to send direct efferents to the hippocampal DL (Atoji et al. 2018). HF tracings in the Garden Warbler revealed strong retrograde connections to HD (Figs. 5b, 7a, c, d, 8), which suggests that our tracer injections in HF included DL. Thereby, our data corroborate previous findings of a direct connection between HA/HD and DM and between HD and DL.

In pigeons, it was shown that HD, in addition to a direct projection to DL, sends afferents to CDL, which, in turn, sends afferents to DM. HD was thus considered to serve as a potential double entry port to HF (Atoji et al. 2005). Our tracings revealed a strong anterograde projection from Cluster N towards the CDL in the Garden Warbler and a corresponding retrograde projection from HF (Figs. 5; 6b, 7b, 8). This confirms both that the tracer was placed into HD, and the existence of a very similar connection in the Garden Warbler forebrain originating from HD to both DL and CDL.

Last, tracer injections into both Cluster N and HF retrogradely labelled the vast majority of neurons within DNH (Figs. 5, 6a, 7a). Those injections will unequivocally have led to labelling of TSM, which originates in HA and runs along the hippocampal surface medially to dive ventrally along the median sagittal plane (Figs. 5, 6a, 7a). This indicates that DNH is connected to TSM and thus represents a previously undescribed HA subdivision.

Taking all shown connectivity together, our neuronal tract tracings in Cluster N included HA, HD and, consequently, all layers in between (i.e. IHA and HI), while HF tracings included DM and DL. Based on these findings, with few, probably species-specific differences, our data largely corroborate the known connectivity described in other bird species. We thereby show that HF and the hyperpallium in the night-migratory Garden Warbler are functionally connected and that the vast majority of hyperpallial connections towards HF originates from HD (Fig. 8a).

As shortly mentioned in the introduction, we used the new avian brain nomenclature proposed by Reiner et al. (2004). Subsequently, several studies that analyzed the transcriptomes of large numbers of zebra finch genes (Chen et al. 2013; Jarvis et al. 2013; Gedman et al. 2021) detected a high level of similarity between HD, HI and MD and proposed a further nomenclature change that reduced the hyperpallial entities to two (HA and IHA) and concomitantly enlarged the mesopallial territory by incorporating HD and HI into MD. This was accompanied by the view of the continuum hypothesis which posits that avian dorsal and ventral pallium wrap around the vestigial lateral ventricle. While we highly value the continuum hypothesis as a useful hypothesis to understand the arrangement of the avian pallium, we see important differences between the connectivity patterns and neurochemical profiles of HD, HI, and MD that speak against a fusion of these areas (Shimizu and Karten 1990; Atoji and Wild 2005, 2012, 2019; Atoji et al. 2018; Kröner and Güntürkün, 1999; Stacho et al. 2020). Based on these findings we outlined our results using the original nomenclature introduced by Reiner et al. (2004).

### A potential pathway for the integration of navigational information

Various studies have suggested a potential involvement of HD in processing navigation-related information: Cluster N activation, which includes HD, has been shown in Garden Warblers (*Sylvia borin*; Mouritsen et al. 2005; Heyers et al. 2007; Hein et al. 2010; this study), European Robins (*Erithacus rubecula*; Liedvogel et al. 2007a; Zapka et al. 2009), Meadow Pipits (*Anthus pratensis*; Zapka et al. 2010), Sardinian Warblers (*Curruca melanocephala*; Liedvogel et al. 2007a) and Northern Wheatears (*Oenanthe oenanthe*; Elbers et al. 2017). This was independently replicated in night-migratory Brownheaded Buntings (*Emberiza bruniceps*) which display high levels of neuronal activation-triggered immediate early genes, in a Cluster N-like visual forebrain structure including HD only at night in their migratory phase (Rastogi et al. 2011).

HD in the thalamofugal pathway, as shown in previous studies (Casini et al. 1986; Kröner and Güntürkün, 1999; Atoji and Wild 2004, 2005; Atoji et al. 2018) seems to originate a major output network to HF either directly to DL, or indirectly via CDL to DM. DM, DL and CDL have been considered the functional backbone of the hippocampal formation in pigeons based on their high synchronization in a study on functional connectivity patterns using blood oxygen level dependent (BOLD) fluctuation analyses (Behroozi et al. 2017). This functional “clustering” could actually be explained by our connectivity results: (1) DM, DL and CDL of Garden Warblers receive their main input from the same source, i.e. HD (Fig. 8a), (2) HD, DM and DL are functionally connected either directly or indirectly via CDL (Figs. 5, 6b, 7b, 8a) and (3) are jointly activated under low light (Figs. 2b, 3b, 4b, g).

Our data indicate that HD represents the main connecting structure between the hyperpallium and the hippocampal DM/DL. Could DM/DL be of central importance in processing navigational information? Indeed, lesion studies indicate that the type of information from the visual pathways reaching the HF is almost certainly not purely visual, since birds with lesioned HF were not impaired on a variety of visual tasks such as delayed matching-to sample, concurrent discrimination or retention of a visual discrimination but rather on spatial tasks (for review, see Colombo and Broadbent 2000). Moreover, a recent study in quails (*Coturnix coturnix*) found direction-sensitive neurons in DM/DL (Ben Yishay et al. 2021). Place cells have been described in the HF of tufted titmice (*Parus bicolor*; Sherry and Hoshooley 2007; Payne et al. 2021). Recent studies in pigeons showed that HF lesions resulted in a complete loss of intensity discrimination while sparing inclination discrimination. In contrast, Wulst lesions had the opposite effect, resulting in a complete loss of inclination discrimination while sparing intensity discrimination (Bingman et al. 2021). This is in agreement with the lesion study of Zapka et al. (2009) showing that the inclination compass but not the magnetic map of a night-migratory songbird is disrupted by Cluster N lesions. Magnetic intensity is thought to be involved in the magnetic map of night-migratory songbirds and requires input from the trigeminal system (Heyers et al. 2010; Kishkinev et al. 2013, 2015; Lefeldt et al. 2014; Elbers et al. 2017; Pakhomov et al. 2018; Kobylkov et al. 2020). Any connections between the trigeminal system and hippocampus, however, are unknown to date.

In addition to magnetic compass information, a very similar role might apply to olfactory navigational information, which is known to be used by navigating pigeons (Gagliardo 2013). The olfactory system connects with HD through reciprocal connections between the olfactory bulbs and the prepiriform and piriform cortices (Bingman et al. 1994; Patzke et al. 2011; Atoji et al. 2014). Homing from familiar or unfamiliar sites increased Egr-1 immunoreactivity in HF, particularly in DM and DL (Shimizu et al. 2004; Patzke et al. 2010).

To sum up, several pieces of evidence indicate that HD could serve as a gateway to HF for navigational (visual, olfactory and magnetic) information in birds. In Garden Warblers, HD appears to represent a central relay, with which magnetic information from the hyperpallium could be transmitted to HF. Within HF, our findings specify DM/DL as a potential hippocampal target, in which visually perceived magnetic compass information could be integrated with other navigational cues in these night-migratory songbirds (Fig. 8b).

## Data Availability

The datasets generated during and/or analysed during the current study are available from the corresponding author upon request.

## Abbreviations

Cb: Cerebellum
CDL: Dorsolateral corticoid area
ClN: Cluster N
DL: Dorsolateral hippocampal subdivision
DLd: Dorsal part of the dorsolateral hippocampal subdivision
DLL: Nucleus dorsolateralis anterior thalami, pars lateralis
DLv: Ventral part of the dorsolateral hippocampal subdivision
DM: Dorsomedial hippocampal subdivision
DMd: Dorsal part of the dorsomedial hippocampal subdivision
DMv: Ventral part of the dorsomedial hippocampal subdivision
DNH: Dorsal nucleus of the hyperpallium
FPL: Fasciculus prosencephali longitudinalis
GLd: Thalamic dorsolateral geniculate complex
H: Hyperpallium
HA: Hyperpallium apicale
HAd: Dorsal part of hyperpallium apicale
HAv: Ventral part of hyperpallium apicale
HD: Hyperpallium densocellulare
HF: Hippocampal formation
HI: Hyperpallium intercalatum
HL: Hyperpallium laterale
IHA: Intercalated part of hyperpallium apicale
LdOPT: Nucleus lateralis dorsalis nuclei optici principalis thalami
LFS: Frontal superior lamina
M: Mesopallium
MD: Mesopallium dorsale
MV: Ventral mesopallium
N: Nidopallium
OT: Optic tectum
Rt: Nucleus rotundus
Tel: Telencephalon
Tr: Triangular hippocampal subdivision
TSM: Tractus septopallio-mesencephalicus
V: V-shaped hippocampal subdivision
Vl: Lateral V-shaped hippocampal subdivision
Vm: Medial V-shaped hippocampal subdivision
